# Sex diversity within U.S. residencies: a cross-sectional study of trends from 2011 to 2019

**DOI:** 10.1186/s12909-022-03565-7

**Published:** 2022-07-05

**Authors:** Ugochi T. Aguwa, Maylander Menard, Divya Srikumaran, Christina Prescott, Joseph Canner, Fasika Woreta

**Affiliations:** 1 Johns Hopkins University School of Medicine, 733 N Broadway, Baltimore, MD 21205 USA; 2 Meharry Medical College School of Medicine, 1005 Dr DB Todd Blvd, Nashville, TN 37208 USA; 3 Wilmer Eye Institute, Johns Hopkins University School of Medicine, 1800 Orleans St, Baltimore, MD 21287 USA; 4 Department of Ophthalmology, NYU Langone Health, 222 E 41st St, New York, NY 10017 USA; 5 Johns Hopkins Surgery Center for Outcomes Research, 733 N Broadway, Baltimore, MD 21205 USA

**Keywords:** Diversity, Sex, Female, Residents, Disparity

## Abstract

**Background:**

Despite females comprising 50.8% of the U.S. population, the percentage of females in the physician workforce is only 36.3%. Studies have examined sex trends within select specialties, however there is insufficient literature studying trends across all specialties. In this study, the authors examined trends in the proportion of female residents from 2011 to 2019 across all specialties, including both surgical and non-surgical.

**Methods:**

Data on the proportion of female residents from 2011 to 2019 in all specialties was extracted from the Accreditation Council for Graduate Medical Education (ACGME) Data Resource Books and analyzed with the chi-square test for trend.

**Results:**

From 2011 to 2019, there was a statistically significant increase in the percentage of female residents in surgical specialties (*p* < 0.001) and no significant change in the percentage of female residents in non-surgical specialties. In the same time period, the specialty with the highest percentage of females was Obstetrics & Gynecology (81.3%), and the specialty with the lowest percentage of females was Orthopedic Surgery (13.8%).

**Conclusions:**

Although there has been a positive overall trend in the percentage of females entering medical and surgical specialties, the percentage of females in medicine overall still lies below that of the entire population. Increased efforts are needed to increase female representation in medicine, especially in the U.S. in specialties where they are traditionally underrepresented.

## Background

It is important that the demographics of physicians represent the demographics of the population. This is not only needed as an equitable norm, but also as a means to improve patient satisfaction and ameliorate health disparities [1]. Yet, a sex disparity exists in medicine, with the percentage of females in medicine (36.3%) in the U.S. falling behind the percentage of females in the entire U.S. population (50.8%) [2, 3]. The Accreditation Council for Graduate Medical Education (ACGME), Liaison Committee on Medical Education (LCME), Association of American Medical Colleges (AAMC), and other governing medical organizations have advocated for increasing diversity in medical education and training in the U.S. [4]. Although the number of female physicians has increased over the years, their proportion still fails to mirror the overall U.S. population, and matters concerning differential, unfair treatment of female physicians must also be confronted.

Previous studies have examined sex diversity trends among residents in select specialties [5–7]. Studies have also explored sex diversity trends in countries outside of the U.S. [8, 9]. However, to our knowledge there is no literature assessing the trends in sex diversity across all specialties in the U.S., specifically comparing surgical specialties to non-surgical specialties. The purpose of this study was to address this gap by investigating trends in the proportion of female residents across various specialties from 2011 to 2019, and examining differences between surgical and non-surgical specialties. The findings from this study can inform and support initiatives to advance female representation and inclusion in all fields of medicine.

## Methods

### Data collection

This study was reviewed and qualified as exempt research by the Johns Hopkins University School of Medicine Institutional Review Board (IRB00266647).

Data for the demographics of all active residents in ACGME residency programs were extracted from ACGME Data Resource Books from 2011 through 2019 [10]. Included categories were: Male, Female, and Not Reported. Importantly, residents self-reported their sex (not gender) for the purposes of these ACGME Data Resource Books. Sex is a static, binary category that corresponds to one's biological classification. The terms male, female, and intersex are used to describe sex [11].

Surgical specialties included in this study were: Neurological Surgery, Obstetrics & Gynecology, Ophthalmology, Orthopedic Surgery, Otolaryngology, Plastic Surgery, Integrated Plastic Surgery, Surgery, Integrated Vascular Surgery, Integrated Thoracic Surgery, and Urology. Non-surgical specialties included: Anesthesiology, Dermatology, Emergency Medicine, Family Medicine, Internal Medicine, Medical Genetics and Genomics, Neurology, Nuclear Medicine, Pathology, Pediatrics, Physical Medicine and Rehabilitation (PM&R), Preventive Medicine, Psychiatry, Radiation Oncology, Radiology, and Internal Medicine/Pediatrics.

### Statistical tests

We used Stata/MP version 14.2 (Stata Corp, College Station, TX) to analyze our data via chi-squared test for trend. Proportions for female residents were defined as the regression of female/(female + male); Δ% represents the slope of chi-squared trend analysis multiplied by 100%. All *P*-values were two-sided. *P*-values ≤ 0.05 were considered statistically significant.

## Results

**Fig. 1:**
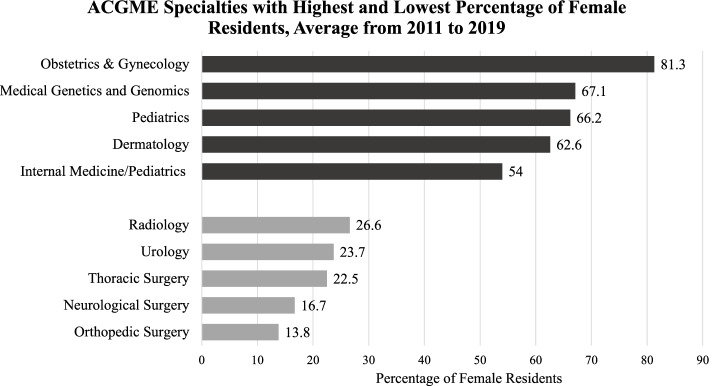
ACGME Specialies with highest and lowest percentage of female residents, average from 2011 to 2019. Abbreviations: *ACGME* Accreditation Council for Graduate Medical Education. Bar graph showing specialties with the highest (black shaded bars) and lowest (grey shaded bars) percentage of female residents. The reported percentage is the average of all percentages within each specialty from 2011 to 2019

Our findings showed that the 5 specialties with the highest average proportion of female residents are: Obstetrics & Gynecology (81.3%), Medical Genetics and Genomics (67.1%), Pediatrics (66.2%), Dermatology (62.6%), and Internal Medicine/Pediatrics (54%). The 5 specialties with the lowest average proportion of female residents were: Orthopedic Surgery (13.8%), Neurological Surgery (16.7%), Integrated Thoracic Surgery (22.5%), Urology (23.7%), Radiology (26.6%) (Fig. 1).

**Fig. 2:**
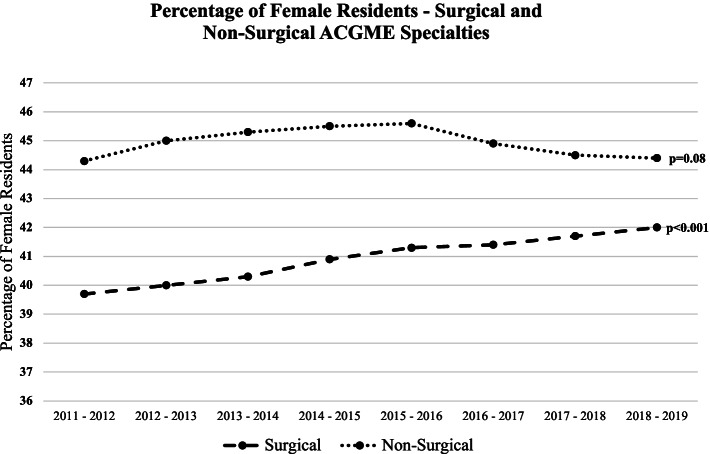
Percentage of female residents – surgical and non-surgical ACGME specialties. Abbreviations: *ACGME* Accreditation Council for Graduate Medical Education. Line graph showing the percentage of female residents by type of specialty (surgical or non-surgical) from 2011 to 2019. The  chi squared test for trend was used to analyze each specialty type and *p*-values can be found to the right of their respective lines; *p*-values ≤ 0.05 were statistically significant

When assessing sex trends between surgical specialties combined and non-surgical specialties combined, there was an overall 2.3% increase in the percentage of female residents in surgical specialties (*p* < 0.001) and no significant change in non-surgical specialties (*p *= 0.08) (Fig. 2). Among female residents in surgical specialties from 2011 to 2019, there was a statistically significant increase in the proportion of female residents in Otolaryngology (*p* = 0.04), Integrated Plastic Surgery (*p* < 0.001), Orthopedic Surgery (*p* = 0.002), Plastic Surgery (*p* = 0.01), Surgery (*p* < 0.001), and Urology (*p* = 0.023); a statistically significant decrease in the proportion of female residents was seen in Ophthalmology (*p* = 0.02). There was a statistically significant increase in the proportion of female residents in the following non-surgical specialties from 2011 to 2019: Internal Medicine (*p* < 0.001) and Neurology (*p* = 0.042); a statistically significant decrease in proportion of female residents was seen in Anesthesiology (*p* = 0.007), Emergency Medicine (*p* = 0.005), Pathology (*p* < 0.001), Pediatrics (*p* = 0.004), and Psychiatry (*p* = 0.003) (Table 1).

**Table 1:** Analysis of trends in the proportion of female residents across all ACGME specialties from 2011 to 2019^a ^

Category	% Change (95% CI)	*P*-Value
Integrated Vascular Surgery	-0.81 (-1.90, 0.28)	0.15
Preventative Medicine	-0.74 (-1.56, 0.08)	0.08
Pathology	-0.69 (-1.01, -0.38)	< 0.001
Ophthalmology	-0.45 (-0.84, -0.06)	0.02
Emergency Medicine	-0.32 (-0.50, -0.14)	0.005
Psychiatry	-0.31 (-0.51, -0.11)	0.003
Radiation Oncology	-0.29 (-0.81, 0.22)	0.26
Anesthesiology	-0.25 (-0.44, -0.07)	0.007
Pediatrics	-0.22 (-0.37, -0.07)	0.004
Diagnostic Radiology	-0.19 (-0.39, 0.001)	0.051
Dermatology	-0.18 (-0.58, 0.21)	0.36
Family Medicine	-0.08 (-0.22, 0.07)	0.30
Obstetrics & Gynecology	0.12 (-0.04, 0.29)	0.138
Physical Medicine & Rehabilitation	0.14 (-0.27, 0.54)	0.51
Orthopedic Surgery	0.27 (0.10, 0.43)	0.002
Internal Medicine/Pediatrics	0.29 (-0.10, 0.69)	0.14
Neurological Surgery	0.30 (-0.01, 0.61)	0.06
Nuclear Medicine	0.30 (-1.13, 1.80)	0.69
Internal Medicine	0.31 (0.21, 0.40)	< 0.001
Neurology	0.31 (0.01, 0.62)	0.042
Otolaryngology	0.38 (0.02, 0.75)	0.04
Urology	0.43 (0.06, 0.80)	0.023
Surgery	0.78 (0.62, 0.94)	< 0.001
Medical Genetics & Genomics	0.80 (-0.87, 2.47)	0.35
Plastic Surgery	1.01 (0.24, 1.78)	0.01
Integrated Plastic Surgery	1.06 (0.45, 1.67)	< 0.001
Integrated Thoracic Surgery	1.10 (-0.24, 2.44)	0.107

*Abbreviations*: *CI* Confidence interval, *ACGME* Accreditation Council for Graduate Medical Education

^a^Data regarding sex demographics of all ACGME residency programs was extracted from ACGME yearly reports 2011 through 2019. The chi-square test for trend was used to analyze the data. *P* ≤ 0.05 were considered statistically significant

## Discussion

Sex diversity remains a problem in medicine, with imbalances between sexes seemingly varying greatly by specialty. Our study shows that the combined percentage of female residents in surgical and non-surgical specialities is less than 45%. These findings align with the established data and highlight a dearth of sex diversity, particularly among surgical specialties in the U.S. Furthermore, recent data showed that the total percentage of female U.S. medical school graduates in 2019 was 47.9% [12]. A possible explanation for this difference (2.9%) could be that some female graduates may not be continuing onto residency. In the U.S., after medical school, the traditional pathway is for medical school graduates to continue to residency. Graduating medical students apply to various programs who then review all potential candidates and select the individuals who they would like to interview. Interviewees can then numerically rank the programs where they are interested in training and, likewise, programs numerically rank the interviewees they would like to train as residents. This extensive process culminates in a computer algorithm sorting applicants to match at the highest residency program that ranked them. The 2.9% difference observed could be a result of factors such as biases in resident selection, decreased exposure during medical school, or preferences of females with respect to the careers they wish to pursue. Future studies should further examine reasons for sex differences, as understanding explanatory factors is an important step in developing actionable measures to reduce disparities.

Our study also showed that Radiology, Urology, Integrated Thoracic Surgery, Neurological Surgery, and Orthopedic Surgery were the five specialties with the lowest percentage of female residents. This coincides with the Association of American Medical Colleges’ (AAMC) 2019 report, which elaborated on the composition of female physicians in academic medicine, and noted Thoracic Surgery, Interventional Radiology, Neurological Surgery, and Orthopedic Surgery as specialties with the lowest percentage of female residents (Urology was not ranked among the lowest, though it was in our study) [13]. These findings also correlate with a few studies that investigated sex diversity trends in specific specialties [5–7]. Shah et al. found that the proportion of female faculty in Orthopedic Surgery grew at a slower rate from 1997 to 2017 as compared to the other specialties they studied [5]. Chapman et al. reported that Diagnostic Radiology ranks 17^th^out of 20 in female representation among the largest residency training programs [6]. All of these studies together with our study uncover a clear need to rectify the sex imbalance across all specialties of medicine. In a study conducted by Tsugawa et al., researchers compared the treatment and outcomes of Medicare patients cared for by female physicians to male physicians and found that patients cared for by female physicians had a lower 30-day mortality and 30-day readmissions rate, even after accounting for potential confounders [14]. This suggests that there is an advantage to increasing female physicians in the medical field because of potential differences in practice patterns between male and female physicians.

Other studies highlighted the diminished sex diversity in academic medicine and leadership, noting that even among specialties with a high number of female physicians entering the field (e.g., Pediatrics and Obstetrics & Gynecology), females are underrepresented as chairs and vice chairs [15]. This trend, has also been observed internationally, as findings from Ramakrishnan et al., who studied female representation in the medical workforce in regions outside the U.S., suggested that even in areas (such as Scandinavia) where female physicians actually comprise a majority of the workforce, they are often underrepresented in positions of leadership [8].

Lewiss et al. explored the bias and implicit systemic inequity females face in academic medicine, which seem to transcend to female physicians in general. Females may lag behind males in key areas of professional development including opportunities to partake in first authorship research publications, research funding, and invitations for lectureship, thus creating barriers to promotion and advancement [15]. The lack of recognition of females in medicine also perpetuates the biases and discrepancies females face in medicine. In a retrospective study by Kuo et al., female general surgery residents were significantly underrepresented as award recipients in comparison to their male counterparts, suggesting the presence of ongoing implicit bias in surgery departments and training programs [16]. In another study by Lin et al. letters of recommendation written for ophthalmology residency applicants demonstrated gender-based differences, particularly in language [17]. This study analyzed 440 applicants (1,318 recommendation letters) who showed no significant difference in USMLE Step 1 score, GPA, number of academic activities, and other factors, yet letters of recommendation written for males, were more “authentic” and contained more “leisure” words and letters of recommendation for females used more “feel” words and “biological process” words; letters of recommendation for female applicants also had fewer adjectives that described abilities such as “analytical” or “genius” [17].

Residency programs ought to make an extended effort to dismantle sex specific bias related to their specialties. Leaders across all specialties should aim to cultivate holistic application reviews and benign training environments for all residency applicants, which may help in the needed female recruitment and retention in medicine. Surgical residencies in particular have high attrition rates among female residents, most often owing to uncontrollable lifestyle and lack of faculty support according to recent studies and survey data [18]. This suggests that malignant training environments in certain specialties relative to others may contribute to decreased female applicants. Lack of female role models may also explain high attrition rates for female residents. We also recognize, however, that some explanation for the varying demographics of female residents in different specialties may reflect female preferences for pursuing (or avoiding) different specialties, as preferences are likely a factor in Obstetrics and Gynecology, for example, where there is female over-representation. Understanding and addressing barriers for females applying in specialties where representation is low, such as surgery, is an important step in increasing sex diversity in medicine.

Alongside improving female recruitment and retention in the medical field, existing disparities must also be confronted. Despite more females than males being enrolled in U.S. medical schools for the first time in history in 2020 [19], disparities in representation and income for practicing physicians remain. The sex wage gap is present in the medical workforce, as female physicians on average earn significantly less than male physicains [20]. In a study by E. Apaydin et al., after adjusting the income of 439 physicians for hours worked per year, specialty, age, years in practice, and other potentially confounding variables, female physicians made on average $27,404 less annually than their male counterparts [21]. A necessary, actionable step towards remedying this wage gap and achieving equity in medicine is drawing attention to this injustice and increasing female representation in senior leadership.

Additionally, sexual harassment of female physicians must be addressed as both a potential cause and consequence of the disparate numbers of females in the medical field. Both inside and outside of medicine, research has found that the likelihood of sexual harassment (and the emotional, physical, and organizational sequalae) increases in occupational settings where the majority of employees are male or if the work being done is commonly regarded work done by males, according to research by Fitzgerald et al. [22]. Sex inequalities endanger the emotional well-being, physical well-being, and organizational well-being of female physicians, since harassment leads to turnover and lower job satisfaction among other consequences. Furthermore, research suggests that discrimination may be a causal factor in the sex disparity in certain specialties, as discrimination may influence a female applicant’s choice of residency [23].

A primary strength of our study was the use of objective data to analyze sex trends across all specialties, thus providing consistent methodology of results and minimizing variability. However, our study is not without limitations. First, some female residents may be included in the "Not Reported" category, which may have affected the accuracy of our results. Additionally, though this study examined the presence of residents self-identifying as female, we acknowledge the importance of representation of non-binary identities in medicine. In the most recent ACGME Data Resource Books (not used in this study), “Non-binary” is now a category alongside “Female”, “Male” and “Not Reported”. This necessary revision is not only more inclusive of all identities but will provide a more comprehensive means to assess the sex diversity of the medical workforce in years to come. In the future, National Resident Matching Program (NRMP) data, which is not publicly available, could be used to provide more direct information on the percent of female residents applying and matching each year, in addition to serving as another useful metric to monitor diversity. Furthermore, a more detailed analysis of how sex demographics in medicine changed as a result of the #MeToo movement, which began in 2017, would offer an interesting perspective.

## Conclusion

This study emphasized the need to increase female recruitment in medicine and highlighted specialties with the greatest sex disparities. Strategic measures should be implemented to better recruit and retain female physicians, especially in surgical specialties. Residency programs should make an extended effort to combat sex specific bias in their specialties and promote training environments that better support female physicians in their field.

## Data Availability

The data that support the findings of this study are publicly available from the Accreditation Council for Graduate Medical Education (ACGME), https://www.acgme.org/about-us/publications-and-resources/graduate-medical-education-data-resource-book/.

## Abbreviations

AAMC: Association of American Medical Colleges
ACGME: Accreditation Council for Graduate Medical Education
AMA: American Medical Association
